# Predictive Biomarkers of Metronomic Chemotherapy Response in Solid Tumors: Chasing an Elusive Signal

**DOI:** 10.3390/cancers18152488

**Published:** 2026-08-03

**Authors:** Piotr Jan Wysocki, Łukasz Kwinta, Ewa Wysocka

**Affiliations:** 1 Department of Oncology, Jagiellonian University-Medical College, 31-501 Krakow, Poland; lkwinta@su.krakow.pl; 2 Department of Oncology, University Hospital, 31-501 Krakow, Poland; 3 Scientific Student Society, Jagiellonian University-Medical College, 31-530 Krakow, Poland

**Keywords:** metronomic chemotherapy, predictive biomarkers, solid tumors, angiogenesis, tumor microenvironment, VEGF, circulating endothelial cells, regulatory T cells, myeloid-derived suppressor cells, gut microbiome, LMIC

## Abstract

Background: Metronomic chemotherapy (MCT), understood as continuous, low-dose cytotoxic administration without prolonged drug-free intervals, has become an established strategy in several solid tumors, acting primarily through antiangiogenic, immunomodulatory, and direct cytostatic mechanisms rather than replication-dependent cytotoxicity. Despite an expanding evidence base, including positive randomized trials, validated predictive biomarkers of response remain unavailable. Methods: We searched PubMed/MEDLINE, Embase, and ClinicalTrials.gov (January 2000 to July 2026) for phase II/III randomized trials, prospective cohorts, and selected retrospective analyses of MCT in breast cancer, head and neck squamous cell carcinoma, NSCLC, and mCRC, and extracted biomarker data from embedded translational substudies of eligible trials. Results: In breast cancer, phase III SYSUCC-001 (adjuvant metronomic capecitabine, improved DFS in TNBC) and MECCA (metronomic capecitabine plus aromatase inhibitor in HR+/HER2− disease) provide the strongest evidence, supported by randomized phase II data for the VEX regimen (METEORA-II) and MCT-anti-PD-1 combinations. TEMPO LUNG established metronomic vinorelbine as effective in platinum-unfit NSCLC, while CAIRO3 confirmed metronomic capecitabine–bevacizumab as an effective mCRC maintenance therapy. Most recently, the phase III TMC-I trial extended positive randomized evidence to head and neck cancer. Candidate biomarkers span angiogenic, immune, tumor proliferative, molecular, pharmacodynamic cytokine, on-treatment clinical (adverse-event-based), and gut–microbiome domains, with FOXC1, circulating endothelial cell kinetics, VEGF pathway markers, and regulatory T-cell dynamics among the most promising; however, none has been prospectively validated in a dedicated confirmatory trial. Conclusions: MCT has moved from empirical use to an evidence-based strategy across multiple tumor types, but the lack of validated predictive biomarkers limits informed patient selection. Future trials should incorporate biomarker-driven designs, particularly FOXC1, endothelial cell kinetics, and immune profiling as co-primary objectives. Defining an MCT-sensitive biological phenotype remains the key translational challenge for the field.

## Simple Summary

Metronomic chemotherapy is a way of giving chemotherapy in small, frequent (even multiple daily) doses instead of the standard high doses given with long breaks in between. It works differently from traditional chemotherapy, mainly by cutting off the blood supply to tumors and by helping the immune system attack cancer cells, rather than simply killing fast-growing cells. This approach has shown real benefit in large clinical trials for breast cancer, lung cancer, colorectal cancer and, most recently, head and neck cancer. However, it is still underused, and doctors currently have no reliable way to predict, before treatment starts, which patients are most likely to respond. This article reviews the existing evidence on biological markers found in blood, tumor tissue, and even gut bacteria that might help identify these responding patients in advance. Markers related to blood vessel growth, immune cell activity, tumor biology, and newer candidates such as certain white blood cells and the composition of the gut microbiome are all discussed. Although several promising signals have been identified, none has yet been formally confirmed in a clinical trial specifically designed to test it. The review concludes by highlighting the most promising candidates and how future studies should be designed to finally answer this open question.

## 1. Introduction

The concept of metronomic chemotherapy (MCT), defined as the continuous or frequent administration of low-dose cytotoxic agents without prolonged drug-free intervals, was formally introduced in 2000 by Browder et al. [1] and Hanahan et al. [2]. This concept built upon earlier observations by Kerbel and colleagues [3], who demonstrated that tumor-associated endothelial cells, unlike malignant cells, are genetically stable and proliferate at a relatively slow but sustained rate. These biological characteristics render endothelial cells susceptible to prolonged antiproliferative and pro-apoptotic stimuli while limiting their ability to develop acquired drug resistance. Consequently, MCT represents a fundamentally different therapeutic paradigm from conventional maximum-tolerated-dose (MTD) chemotherapy, which is designed primarily to eliminate rapidly dividing tumor cells. Rather than relying predominantly on direct cytotoxicity, MCT exerts its antitumor activity through several complementary mechanisms, including: (i) sustained inhibition of tumor angiogenesis mediated by endothelial cell apoptosis and upregulation of the endogenous angiogenesis inhibitor thrombospondin-1 (TSP-1); (ii) immunomodulation through the selective depletion of immunosuppressive regulatory T cells (Tregs), thereby restoring endogenous antitumor immunity; and (iii) direct cytostatic effects resulting from prolonged exposure of tumor cells to low drug concentrations. Because these mechanisms differ fundamentally from those underlying conventional chemotherapy, predicting response to MCT is also likely to require a distinct biomarker framework.

Several clinical characteristics make MCT an attractive therapeutic strategy, particularly in settings where conventional intensive chemotherapy is either poorly tolerated or not clinically indicated. Its predominantly oral administration, favorable toxicity profile, and limited need for hospital-based treatment make MCT especially suitable for patients with low-volume or indolent disease. These practical advantages are equally relevant for elderly or frail patients, individuals living in rural areas, and populations in low- and middle-income countries (LMICs), where access to infusion-based therapies may be limited. Consequently, MCT offers not only a biologically distinct treatment approach but also a pragmatic option for improving access to systemic anticancer therapy.

Although MCT has been investigated for more than two decades, its clinical evidence base was, until recently, dominated by single-arm phase II studies and retrospective analyses. This landscape has changed substantially over the past five years with the publication of several pivotal randomized trials, including the phase III SYSUCC-001 study in early triple-negative breast cancer, the phase III MECCA trial in hormone receptor-positive metastatic breast cancer, the phase III TMC-1 trial in head and neck squamous cell carcinoma, as well as the randomized phase II METEORA-II study in metastatic breast cancer. Together, these studies have considerably strengthened the clinical evidence supporting MCT and have shifted its role from an experimental concept toward an evidence-based therapeutic strategy. Furthermore, MCT has demonstrated clinical activity both as monotherapy and as a backbone for combination treatment across multiple tumor types and clinical settings [4,5,6,7,8,9].

Despite these advances, the identification and prospective validation of predictive biomarkers for MCT response remain major unresolved challenges in the field. In current clinical practice, patient selection is largely based on indirect clinical characteristics, such as treatment refractoriness, performance status, or expected tolerability, rather than on tumor biology. This approach is unlikely to be optimal because MCT and conventional MTD chemotherapy probably benefit biologically distinct patient populations. Highly proliferative tumors, which are particularly susceptible to replication-dependent cytotoxicity induced by MTD chemotherapy, may derive relatively limited benefit from therapies acting predominantly through antiangiogenic and immunomodulatory mechanisms. In contrast, slowly proliferating, well-differentiated tumors embedded within an immunosuppressive and angiogenesis-dependent microenvironment may be inherently more responsive to MCT. Defining the biological characteristics of this MCT-sensitive phenotype, rather than relying solely on clinical surrogates, therefore represents the central translational challenge this review addresses.

Much of this unresolved status reflects methodological limitations of the biomarker literature itself, rather than an absence of biologically plausible candidates. Most published analyses are small, single-institution, exploratory correlative sub-studies embedded within trials that were neither designed nor powered to test a biomarker hypothesis. Therefore, positive findings are frequently unreplicated, and negative findings are rarely reported at all. Heterogeneity in MCT regimens across studies, discussed further in Section 2, compounds this problem by making cross-trial comparisons of biomarker performance difficult.

This review aims to: (1) summarize the current clinical evidence supporting MCT across four solid tumor types in which it has been most extensively investigated—breast cancer, head and neck squamous-cell cancer, non-small cell lung cancer, and colorectal cancer; (2) systematically evaluate candidate predictive biomarkers of treatment response; (3) identify the mechanistic pathways that appear most relevant to MCT sensitivity; and (4) propose a conceptual framework to guide the design of future biomarker-driven clinical trials.

## 2. Mechanisms of Metronomic Chemotherapy: Implications for Biomarker Selection

A thorough understanding of the mechanisms underlying the antitumor activity of metronomic chemotherapy (MCT) is essential for identifying clinically meaningful predictive biomarkers. In contrast to maximum-tolerated-dose (MTD) chemotherapy, whose efficacy depends largely on direct cytotoxic effects such as DNA damage or mitotic spindle disruption in rapidly proliferating cells, MCT targets multiple biological processes that are primarily regulated by the tumor microenvironment (TME) rather than by the intrinsic characteristics of malignant cells [10,11,12,13,14]. Although these mechanisms are closely interconnected, three principal pathways account for the therapeutic activity of MCT, and their relative contribution likely varies according to tumor biology, treatment regimen, and disease setting. Much of the mechanistic evidence supporting these pathways, in particular the immunomodulatory and cytostatic effects described below, remains preclinical and has not been directly confirmed in patients treated with MCT.

### 2.1. Anti-Angiogenesis

The antiangiogenic effect of MCT, first established by Kerbel and colleagues, remains the cornerstone of its biological rationale. Continuous exposure to low concentrations of cytotoxic agents selectively targets proliferating endothelial cells within the tumor vasculature while largely avoiding the hematological toxicity associated with conventional MTD chemotherapy. This effect has been demonstrated most consistently for alkylating agents such as cyclophosphamide (CTX), although similar activity has also been reported for several antimetabolites. In parallel, MCT promotes the expression of thrombospondin-1 (TSP-1), a potent endogenous inhibitor of angiogenesis, further reinforcing the suppression of tumor neovascularization [15]. In addition, sustained treatment reduces the mobilization of circulating endothelial progenitor cells (CEPs) from the bone marrow, thereby limiting their contribution to vascular remodeling and new vessel formation.

These biological effects provide a clear rationale for several candidate predictive biomarkers, including baseline microvessel density, circulating concentrations of VEGF and TSP-1, and treatment-induced changes in circulating endothelial cells (CECs) and CEPs. At first glance, highly vascularized, VEGF-dependent tumors might be expected to derive the greatest benefit from antiangiogenic MCT. However, available evidence indicates that this relationship is considerably more complex. Clear cell renal cell carcinoma (ccRCC), one of the most angiogenesis-dependent solid tumors, represents an instructive example [16]. Despite its profound dependence on VEGF signaling, MCT has not been developed in ccRCC as an antiangiogenic strategy; instead, clinical studies have focused almost exclusively on its immunomodulatory properties, particularly the depletion of regulatory T cells. Furthermore, therapies directly targeting the VEGF/VEGFR axis have failed to identify VHL mutation status or baseline VEGF levels as reliable predictive biomarkers in ccRCC [17,18]. These observations suggest that activation of the VEGF pathway alone is insufficient to define sensitivity to MCT. Rather, responsiveness is likely determined by the interplay among angiogenesis, immune composition, stromal architecture, and compensatory pro-angiogenic pathways. Conversely, tumors characterized by intrinsically low vascular density and relative independence from angiogenesis may be inherently less responsive to the antiangiogenic component of MCT.

### 2.2. Immunomodulatory Mechanism

Beyond its vascular effects, MCT exerts profound immunomodulatory activity that may represent its most therapeutically exploitable mechanism. Among these effects, the selective depletion of CD4+CD25+FoxP3+ regulatory T cells (Tregs) has attracted particular attention because of the central role of these cells in maintaining tumor-induced immune tolerance [19]. Reducing Treg numbers within the TME restores the activity of cytotoxic CD8+ T lymphocytes and enhances endogenous antitumor immune responses. Low-dose CTX administered on a metronomic schedule preferentially targets Tregs while largely sparing effector T cells, a selectivity that is not observed with conventional MTD administration. This differential sensitivity has been attributed to the continuous, low-level proliferation of Tregs, which renders them particularly susceptible to persistent DNA damage.

These observations suggest that the baseline immune composition of the TME may influence the clinical response to MCT. In particular, the Treg-to-CD8 ratio and the density of tumor-infiltrating lymphocytes (TILs) represent plausible predictive biomarkers. Tumors enriched in Tregs but relatively depleted of effector T cells—the so-called immunologically “cold” phenotype—may be especially susceptible to immune reprogramming induced by MCT. This concept also provides the biological rationale for combining MCT with immune checkpoint inhibitors (ICIs), in which metronomic therapy is intended to remodel the immune microenvironment prior to checkpoint blockade, as discussed in Section 3.5.

### 2.3. Direct Cytostatic and Anti-Tumor Cell Effects

In addition to its antiangiogenic and immunomodulatory properties, MCT exerts direct antitumor activity through sustained exposure of malignant cells to subcytotoxic drug concentrations. Rather than inducing extensive tumor cell death, prolonged low-dose treatment promotes cell-cycle arrest, cellular senescence, and suppression of tumor repopulation during periods that would otherwise permit recovery between cycles of conventional chemotherapy. 

An important consequence of this cytostatic mechanism concerns the relationship between tumor proliferative activity and sensitivity to MCT. High Ki67 expression is a well-established predictor of response to conventional MTD chemotherapy, reflecting the dependence of these regimens on active tumor cell proliferation. The situation may be fundamentally different for MCT. Tumors with low proliferative activity, which typically respond poorly to conventional cytotoxic therapy, may instead possess precisely the biological characteristics that favor metronomic treatment, including an immunosuppressive microenvironment, increased Treg infiltration, and greater dependence on angiogenesis. This apparent proliferative paradox—the hypothesis that MCT and MTD chemotherapy preferentially benefit biologically distinct tumor subtypes defined by their proliferative phenotype—provides a compelling mechanistic framework for biomarker development and is examined further in the clinical context in Section 4.

**Figure 1:**
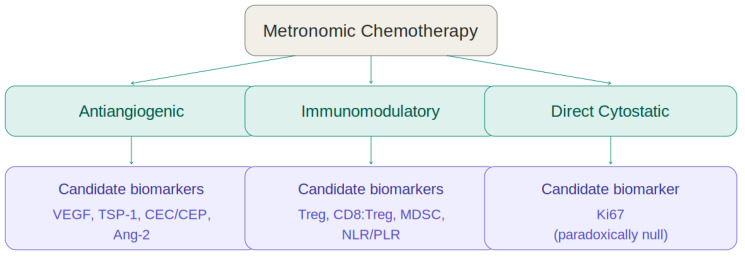
The three principal mechanisms of metronomic chemotherapy and their associated candidate predictive biomarkers.

Two points of caution apply when translating these mechanisms into biomarker hypotheses. First, MCT is not a single, uniform intervention: published regimens vary widely in the agent used, dose, schedule, combination partner, and treatment line, and this heterogeneity is a plausible source of the inconsistent biomarker findings discussed in Section 4. Second, each mechanistic pathway points toward a distinct family of candidate biomarkers, like circulating endothelial cells and VEGF-pathway markers for the antiangiogenic effect; regulatory T-cell and myeloid-derived suppressor cell dynamics for the immunomodulatory effect; and proliferative indices such as Ki67 for the cytostatic effect. Together, these provide the organizing framework for the biomarker domains reviewed below (Figure 1).

## 3. Clinical Evidence Base: Phase II and Phase III Trials

### 3.1. Breast Cancer

#### 3.1.1. Triple-Negative Breast Cancer

The most mature phase III evidence supporting MCT in any solid tumor comes from the SYSUCC-001 trial (NCT01112826). This randomized phase III study enrolled 443 patients with operable triple-negative breast cancer (TNBC) who had completed standard adjuvant chemotherapy [20]. Participants were assigned to either one year of metronomic capecitabine or observation. After a median follow-up of 56.5 months, 5-year disease-free survival (DFS) was significantly higher in the MCT group than in the observation group. An updated 10-year analysis, conducted after a median follow-up of 116 months, confirmed the durability of the DFS benefit [21]. Ten-year DFS was 78.1% in the capecitabine group and 66.6% in the observation group (HR 0.61, 95% CI 0.43–0.88; *p* = 0.0074). Ten-year OS was numerically higher with metronomic capecitabine, although the difference remained below the threshold for statistical significance (82.4% vs. 73.7%; HR 0.67; *p* = 0.058). This analysis also included a post hoc exploratory assessment of FOXC1 expression by immunohistochemistry in baseline tumor samples, representing the first molecular biomarker dataset derived from a phase III MCT trial. These findings are discussed in detail in Section 4.4.

In metastatic TNBC, MCT has been evaluated mainly in combination with immune checkpoint inhibitors. A Bayesian adaptive randomized phase II trial [22] compared five regimens in patients with HER2-negative metastatic breast cancer, including both TNBC and HR-positive/HER2-negative disease, who had received no more than one previous line of chemotherapy. The treatment groups comprised metronomic vinorelbine (VNR) monotherapy (*n* = 11), VNR plus toripalimab (*n* = 7), bevacizumab plus VNR and toripalimab (*n* = 27), conventional cisplatin plus VNR and toripalimab (*n* = 26; control arm), and metronomic cyclophosphamide plus capecitabine, VNR, and toripalimab (VEX cohort; *n* = 26). The primary endpoint was disease control rate (DCR), with progression-free survival (PFS) and safety included as secondary endpoints. The metronomic VEX cohort achieved a DCR of 69.7% and the longest median PFS among all treatment groups, at 6.6 months, compared with 3.5 months in the conventional cisplatin arm. Exploratory biomarker findings from this study are reviewed in Section 4.2.

#### 3.1.2. Hormone Receptor-Positive/HER2-Negative Breast Cancer

Two pivotal studies, MECCA and METEORA-II, evaluated MCT in hormone receptor-positive/HER2-negative breast cancer. In the phase III MECCA trial [23], 263 patients with previously untreated HR-positive/HER2-negative metastatic breast cancer were randomized to receive metronomic capecitabine plus an aromatase inhibitor (AI) or an AI alone. The combination significantly improved both PFS (HR = 0.58 [95% CI, 0.43 to 0.76], with a median PFS of 20.9 months for AI+capecitabine compared with 11.9 months for AI alone) and OS (HR = 0.58 [95% CI, 0.37 to 0.93]), supporting metronomic capecitabine as an active first-line chemotherapy option for patients who cannot tolerate or do not have access to CDK4/6 inhibitors. No biomarker analyses were reported.

The randomized phase II METEORA-II trial [24] enrolled 140 patients with ER-positive/HER2-negative metastatic breast cancer and compared the fully oral metronomic VEX regimen (vinorelbine 40 mg three times weekly, cyclophosphamide 50 mg daily, and capecitabine 500 mg twice daily) with weekly intravenous paclitaxel. VEX produced significantly better outcomes for both time to treatment failure (8.3 vs. 5.7 months; HR 0.61, 95% CI 0.42–0.88; *p* = 0.008) and PFS (11.1 vs. 6.9 months; HR 0.67, 95% CI 0.46–0.96; *p* = 0.03). The DCR was numerically higher with VEX (68.6% vs. 55.6%), although the difference was not statistically significant. OS was also similar between groups (29.5 vs. 33.7 months; HR 0.98; *p* = 0.90). The METEORA-II regimen is conceptually similar to the FulVEC regimen, which consists of fulvestrant plus VEC(X) and was evaluated in our clinical program in a heavily pretreated, treatment-refractory population [4]. In that prospectively collected cohort of 72 patients, FulVEC demonstrated meaningful clinical activity despite extensive prior treatment, with non-progression rates ranging from 73.2% in the lowest adverse-event stratum to 91.7% in the highest. Ki67 showed no predictive value for any efficacy endpoint, whereas the occurrence and severity of treatment-emergent adverse events emerged as a promising pharmacodynamic signal of clinical benefit, discussed in detail in Section 4.6.

Further prospective phase II evidence comes from the GOIM 21003 trial [25] and the METRO trial [26], which evaluated metronomic doxorubicin plus CTX and metronomic capecitabine plus CTX, respectively, across different lines of therapy in advanced breast cancer. The Italian VICTOR-6 study [27] additionally provided real-world evidence from 112 patients aged 75 years or older, with a median age of 81 years. An objective response rate (ORR) of 27.9% and a DCR of 79.3% were observed, supporting MCT as a feasible option for elderly and frail patients with metastatic breast cancer, a population that remains systematically underrepresented in clinical trials.

### 3.2. Non-Small-Cell Lung Cancer

The clinical evidence supporting MCT in non-small cell lung cancer (NSCLC) is considerably less mature than that available in breast cancer, and no phase III trial has yet been completed. The current evidence base consists of one randomized phase II study, several prospective non-randomized cohort studies, and an individual patient data meta-analysis.

TEMPO LUNG [28] remains the only randomized trial of MCT in NSCLC. This multicenter, open-label phase II study enrolled 167 patients with stage IIIB–IV NSCLC who were considered unfit for platinum-based combination chemotherapy. Participants were randomized to metronomic oral vinorelbine at a fixed dose of 50 mg three times weekly or to a conventional weekly vinorelbine schedule of 60–80 mg/m^2^. The primary endpoint was PFS without grade 4 or higher toxicity (G4PFS). Metronomic vinorelbine significantly prolonged median G4PFS (4.0 vs. 2.2 months; HR 0.63, 95% CI 0.45–0.88; *p* = 0.007) and was associated with substantially fewer grade 3–4 treatment-related adverse events (25.3% vs. 54.4%). This difference was driven mainly by a marked reduction in grade 3–4 neutropenia (10.8% vs. 42.0%). Conventional PFS did not differ significantly between groups (4.3 vs. 3.9 months), and no OS benefit was observed. Quality-of-life scores were comparable. These findings support metronomic oral vinorelbine as a treatment option for platinum-unfit patients, including older individuals and those with poor performance status.

The prospective phase II MOVE trial [29] enrolled 43 chemotherapy-naive patients aged 70 years or older with stage IIIB–IV NSCLC. The median age was 80 years, and more than half of the participants had a performance status of 2. Patients received metronomic oral vinorelbine at 50 mg three times weekly until disease progression or unacceptable toxicity. The co-primary endpoints were ORR, clinical benefit rate (CBR; objective response or stable disease lasting more than 12 weeks), and safety. A CBR of 58.1% and an ORR of 18% were observed. Median time to progression was 5 months, and median OS was 9 months. Grade 3–4 treatment-related adverse events occurred in 11% of patients, with no treatment-related deaths. Serial measurements of serum VEGF and TSP-1 were performed in a subgroup, yielding early pharmacodynamic data discussed in Section 4.1.

Another phase II study [30] evaluated metronomic oral vinorelbine, administered at 50 mg three times weekly during induction and reduced to 30 mg during concurrent radiotherapy, in combination with cisplatin 80 mg/m^2^ every three weeks. The regimen was used as induction therapy followed by concurrent chemoradiotherapy to 66 Gy in 65 treatment-naive patients with unresectable stage III NSCLC. Outcomes were comparable to historical results from standard-dose regimens, with an ORR of 66.2%, a median PFS of 11.5 months, a median OS of 35.6 months, and a favorable toxicity profile. An exploratory analysis of circulating tumor DNA (ctDNA) identified baseline liquid biopsy status as a potential response biomarker, as discussed further in Section 4.

An individual patient data meta-analysis [31] pooled nine non-comparative phase II studies of metronomic oral vinorelbine in metastatic NSCLC. Fixed doses of 30–50 mg were administered three times weekly to a total of 418 patients, 80% of whom had features of frailty. Median OS was 8.7 months (95% CI 7.6–9.5), median PFS was 4.2 months (95% CI 3.9–5.0), and the 6-month OS rate was 64%. Grade 3–4 toxicity occurred in 15.8% of patients, while anemia was the most common adverse event overall. In multivariable Cox models, performance status 2 and stage IV disease independently predicted shorter OS and PFS. Although the analysis confirmed consistent activity and tolerability across studies in a predominantly frail population, the lack of randomized comparators and the non-comparative design of all included trials prevent firm conclusions regarding efficacy.

### 3.3. Metastatic Colorectal Cancer

The role of MCT in metastatic colorectal cancer (mCRC) differs from that in breast cancer and NSCLC. Its most established application is as maintenance therapy after induction chemotherapy, although more recent studies have also explored MCT as part of combination salvage strategies in microsatellite-stable/proficient mismatch repair (MSS/pMMR) disease.

The CAIRO3 trial [32] randomized 558 previously untreated patients with mCRC who had achieved at least stable disease after six cycles of capecitabine, oxaliplatin, and bevacizumab (CAPOX-B). Patients were assigned to receive maintenance capecitabine administered on a metronomic, daily schedule plus bevacizumab, or to observation. CAPOX-B was reintroduced after first progression in both groups. The primary endpoint, second PFS (PFS2), was significantly prolonged with maintenance therapy (11.7 vs. 8.1 months; HR 0.67; *p* = 0.0002). First PFS was also improved (8.5 vs. 4.1 months; HR 0.44; *p* < 0.0001), whereas the numerical OS advantage did not reach statistical significance (21.6 vs. 18.1 months; HR 0.89; *p* = 0.22). Maintenance treatment did not adversely affect quality of life. CAIRO3 remains the only completed phase III randomized trial of MCT in mCRC and supports metronomic capecitabine plus bevacizumab as a standard maintenance option following oxaliplatin-based induction.

The Epitopes-CRC01 trial [33] is the only prospective study specifically designed to investigate biomarkers of MCT response in mCRC. Seventy-seven heavily pretreated patients with MSS/pMMR disease received multimodal MCT consisting of continuously administered capecitabine, cyclophosphamide, and aspirin. Plasma and peripheral blood mononuclear cells were collected prospectively at baseline and during treatment. Median PFS was 2.8 months, 28 patients (36%) remained progression-free for more than 3 months, and 15 (19%) for more than 6 months. These results indicate modest but potentially meaningful activity in a chemorefractory mCRC population, with no new safety signals. Liver metastases were present in 69.4% of non-responders but in only 28.6% of patients with PFS exceeding 3 months (*p* < 0.001), suggesting a possible clinical selection factor. The translational biomarker findings are discussed in Section 4.

A retrospective study [34] evaluated later-line treatment with metronomic capecitabine combined with a VEGFR2 tyrosine kinase inhibitor and a PD-1 inhibitor in MSS/pMMR mCRC. Fourteen patients received fruquintinib, camrelizumab, and metronomic capecitabine, whereas seven received anlotinib, tislelizumab, and metronomic capecitabine. After propensity score matching with 42 patients treated with standard later-line regimens, the triplet strategy produced an ORR of 33.3%, a DCR of 90.5%, a median PFS of 5.4 months, and a median OS of 10.4 months. These findings are notable given the well-established resistance of MSS/pMMR mCRC to ICI monotherapy, with historical response rates below 5%. The results prompted the initiation of an ongoing phase II trial (NCT06148402) and provide preliminary evidence that MCT-mediated remodeling of the tumor microenvironment (TME) may help overcome the immune exclusion characteristic of MSS colorectal tumors.

An ongoing randomized controlled trial [35] is evaluating metronomic, daily capecitabine at 500 mg/m^2^ twice daily, administered continuously for two years, against watchful waiting in 240 patients with resected/ablated stage IV mCRC and an Eastern Cooperative Oncology Group performance status of 0–1. Eligible patients must have achieved no evidence of disease after R0 resection or complete ablation of the primary tumor and all hepatic and/or pulmonary metastases, followed by completion of perioperative chemotherapy. The primary endpoints are DFS and OS, with safety and quality of life included as secondary outcomes. The study addresses two clinically relevant gaps: current colorectal cancer guidelines provide no specific treatment recommendation for patients with mCRC who achieve no evidence of disease, and the role of MCT in this setting remains undefined.

### 3.4. Other Solid Tumors

Beyond breast cancer, NSCLC, and mCRC, MCT has been investigated in several other solid tumors. These studies help define the broader clinical relevance of its antiangiogenic, immunomodulatory, and cytostatic mechanisms.

The most compelling evidence comes from head and neck squamous cell carcinoma (HNSCC). A randomized phase III trial [36], presented as a Late-Breaking Abstract at the 2026 ASCO Annual Meeting, enrolled 422 patients with platinum-sensitive, treatment-naive recurrent or metastatic HNSCC. Participants were randomized to MTD chemotherapy consisting of paclitaxel plus carboplatin administered every three weeks or to the TMC-I regimen, comprising oral metronomic methotrexate 9 mg/m^2^ weekly, celecoxib 200 mg twice daily, erlotinib 150 mg daily, and ultra-low-dose nivolumab 20 mg intravenously every three weeks. Median OS, the primary endpoint, was 10.3 months with TMC-I and 6.2 months with chemotherapy (HR 0.57, 95% CI 0.44–0.73; *p* < 0.0001), corresponding to a 43% reduction in the risk of death. Six-month and 1-year OS rates were 69.2% vs. 52.3% and 46.4% versus 23.2% for the TMC-I regimen and standard chemotherapy, respectively. Secondary endpoints also favored TMC-I. The ORR was 53.4% versus 24.1% (*p* < 0.001), median PFS was 5.5 versus 2.7 months (HR 0.47, 95% CI 0.37–0.58; *p* < 0.001), and the 1-year PFS rates were 26.1% and 4.7%, respectively. Median duration of response was 11.0 months with TMC-I and 3.6 months with chemotherapy (HR 0.32, 95% CI 0.19–0.53). No treatment-related deaths were reported. Patient-reported quality of life was preserved with TMC-I, and grade 3 or higher adverse events were less frequent with MCT than with standard chemotherapy (34.1% vs. 46.4%; *p* = 0.010). The toxicity profiles also differed: liver enzyme elevation, hyperbilirubinemia, and rash were more characteristic of TMC-I, whereas hematological toxicity predominated in the chemotherapy group.

The estimated monthly cost of TMC-I, approximately $230 compared with more than $1000 for standard regimens, may be particularly relevant in low- and middle-income countries, where access to platinum-based chemotherapy, immunotherapy, and cetuximab remains limited. Interpretation of the study is constrained by the use of a comparator that did not include pembrolizumab combined with paclitaxel and carboplatin. Nevertheless, the trial provides the first robust phase III randomized evidence of a clinically meaningful benefit from an MCT-based strategy in HNSCC. The inclusion of ultra-low-dose nivolumab also establishes TMC-I as an MCT–ICI combination, the immunological rationale and biomarker implications of which are discussed in Section 3.5.

In recurrent ovarian cancer, another highly angiogenesis-dependent tumor type, metronomic oral cyclophosphamide combined with bevacizumab produced response rates of up to 42% in a prospective cohort of 70 patients with platinum-refractory disease [37]. These findings support the antiangiogenic rationale for MCT in VEGF-dependent gynecological malignancies. Metronomic topotecan-based regimens, including topotecan combined with cyclophosphamide (CyTo), have also shown clinical activity in heavily pretreated advanced ovarian cancer [38], supporting the use of MCT across multiple lines of therapy in this setting.

In castration-resistant prostate cancer, several phase II trials have reported activity with metronomic regimens based on cyclophosphamide, vinorelbine, etoposide, or methotrexate. This disease setting also generated the only formal pharmacogenomic dataset for MCT, as discussed in Section 4.1.

Hepatocellular carcinoma represents another relevant example because of its pronounced vascularity. A phase II trial [39] of multimodal MCT with metronomic capecitabine, pioglitazone, and rofecoxib reported a median PFS of 8.4 months among patients who remained on treatment for at least six weeks and a median OS of 9.4 months in the same subgroup. A reduction in C-reactive protein was associated with improved OS, providing an early signal that inflammatory markers may have prognostic or predictive relevance in MCT-treated hepatocellular carcinoma.

Across these tumor types, MCT has shown activity in settings characterized by angiogenic dependence, immune suppression, and treatment resistance, broadly consistent with its proposed mechanisms of action. However, apart from HNSCC, randomized phase III evidence remains lacking. This limitation underscores both the clinical opportunity and the need for biomarker-guided strategies to identify the tumor types and patient subgroups most likely to benefit.

### 3.5. MCT as an Immunomodulatory Backbone for ICI Combinations

The rationale for combining MCT with immune checkpoint inhibitors rests primarily on the ability of low-dose cyclophosphamide to selectively deplete regulatory T cells. By reducing immunosuppressive pressure within the TME, MCT may help convert immunologically “cold” tumors into a state that is more responsive to checkpoint blockade. This concept has supported the development of MCT–ICI combinations in breast cancer, HNSCC, MSS/pMMR mCRC, and renal cell carcinoma.

In breast cancer, the most informative evidence comes from the Bayesian adaptive randomized phase II trial in metastatic TNBC described in Section 3.1.1. The metronomic VEX cohort achieved a DCR of 69.7% and a median PFS of 6.6 months, the longest among all study arms. Importantly, clinical benefit correlated with immune reprogramming during treatment rather than with baseline immune parameters. This observation supports the proposed immunomodulatory role of MCT and suggests that dynamic, on-treatment immune monitoring may be more informative than pretreatment profiling in this context.

In HNSCC, the phase III LBA6007 trial incorporated ultra-low-dose nivolumab as an integral component of the TMC-I regimen alongside three orally administered metronomic agents. The substantial OS benefit observed in this study suggests that MCT-mediated remodeling of the TME may enhance the activity of checkpoint blockade even when nivolumab is administered at a fraction of its conventional dose.

In MSS/pMMR mCRC, a tumor subtype generally considered resistant to immunotherapy, metronomic capecitabine combined with a VEGFR2 tyrosine kinase inhibitor and a PD-1 inhibitor produced a clinically relevant efficacy signal and led to the initiation of an ongoing phase II trial (NCT06148402). The proposed mechanism is that MCT-mediated Treg depletion and VEGFR2-mediated antiangiogenic remodeling jointly alter the immunosuppressive microenvironment, thereby facilitating responsiveness to checkpoint blockade. Confirmation of this hypothesis would introduce a potentially important strategy for a disease subtype with limited later-line therapeutic options.

In renal cell carcinoma, the phase Ib CAPER trial [40] is evaluating a 21-day metronomic cyclophosphamide run-in followed by combined cyclophosphamide and pembrolizumab in patients with clear-cell metastatic RCC who have progressed after prior ICI therapy. The biological premise is that metronomic CTX may deplete treatment-resistant Tregs that have reaccumulated after anti-PD-1 or anti-PD-L1 exposure, thereby restoring sensitivity to checkpoint blockade. Serial biopsies obtained at baseline, after the MCT run-in, and during combination treatment will allow longitudinal assessment of Treg dynamics and other mechanistic biomarkers.

A consistent therapeutic pattern is therefore emerging across breast cancer, HNSCC, MSS/pMMR mCRC, and RCC. In these settings, MCT is used less as the principal cytotoxic component than as an immunological primer intended to modify the TME and enable or restore sensitivity to ICIs. MCT–ICI combinations should consequently be regarded as a distinct therapeutic strategy rather than as a simple extension of either MCT or ICI monotherapy. Their biomarker requirements are also likely to differ. Relevant signals may include baseline or treatment-induced changes in Treg density, the CD8 ratio, or myeloid-derived suppressor cell frequency, rather than conventional markers such as PD-L1 expression or tumor mutational burden. This interpretation is supported by the Mo et al. trial [22], in which none of the baseline immune parameters predicted clinical benefit, and is consistent with the absence of PD-L1-based enrichment in the MCT-I trial [36].

Taken together, the clinical evidence summarized in Table 1 shows that MCT is now supported by positive phase III data in four solid tumor types—breast cancer, NSCLC, mCRC, and, most recently, HNSCC—with consistent, moderate-to-large treatment effects despite considerable heterogeneity in trial design. None of these trials, however, was designed or powered to determine which patients within each population derive that benefit. The biomarker evidence reviewed in the remainder of this article comes almost entirely from exploratory, non-prespecified sub-studies embedded within these and smaller phase II trials and should be interpreted accordingly.

## 4. Predictive Biomarkers of MCT Activity

**Figure 2:**
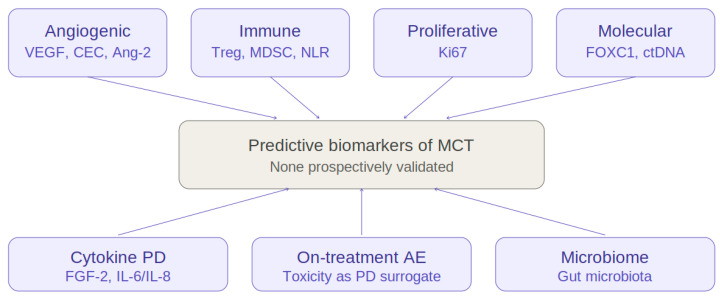
Potential biomarkers for metronomic chemotherapy response.

Despite several decades of clinical investigation, no biomarker of MCT response has yet been prospectively validated in a confirmatory randomized trial. Available evidence comes exclusively from exploratory analyses embedded within clinical studies, translational investigations based on single cohorts, or retrospective series. The candidate biomarkers can be grouped into seven mechanistically distinct domains: angiogenic, immune, tumor proliferative, molecular, pharmacodynamic cytokine, on-treatment clinical (adverse event-based), and gut microbiome (Figure 2). These biomarkers are summarized, together with their associated tumor types, directions of effect, and levels of evidence, in Table 2.

### 4.1. Angiogenic Biomarkers

Because inhibition of angiogenesis is a central mechanism of MCT, markers of tumor vascularity and VEGF pathway activity have received the greatest attention. VEGF-A, thrombospondin-1 (TSP-1), circulating endothelial cells (CECs), and circulating endothelial progenitor cells (CEPs) have all been investigated as potential predictive or pharmacodynamic biomarkers [53].

The most clinically informative evidence comes from a prospective cohort of 46 patients with advanced breast cancer treated with metronomic cyclophosphamide, capecitabine, and bevacizumab [41]. Baseline CEC levels above the first quartile were independently associated with a longer time to progression (*p* = 0.021). In addition, VEGF-A concentrations measured after two months of treatment were significantly lower among the 15 long-term responders, defined as those remaining progression-free for more than one year, than among non-responders. At disease progression, CEC levels declined, while VEGF-A and basic fibroblast growth factor (bFGF) increased, suggesting a shift in the vascularization phenotype as a potential mechanism of resistance. These findings support baseline CECs as candidate predictive biomarkers and on-treatment VEGF-A as a possible pharmacodynamic monitoring tool.

In the MOVE trial of elderly patients with NSCLC, non-responders had lower baseline VEGF levels followed by a rapid increase during treatment [29]. This inverse pattern may reflect compensatory upregulation of VEGF in tumors that are relatively independent of angiogenesis at baseline. TSP-1 levels remained unchanged during treatment in both responders and non-responders, arguing against its usefulness as an on-treatment pharmacodynamic marker.

A separate pharmacodynamic study of metronomic vinorelbine conducted by the Hellenic Cooperative Oncology Group in a mixed solid tumor population identified baseline FGF-2 and IL-8 as significant predictors of a favorable response, whereas baseline VEGF did not differ between responders and non-responders [54]. This result contrasts with the MOVE findings. The discrepancy may be partly explained by differences in tumor type, as MOVE included only NSCLC, whereas the Hellenic study enrolled patients with several solid malignancies. More importantly, it illustrates that the angiogenic biomarker profile of MCT is unlikely to be captured by a single marker and may vary according to tumor context. Future prospective studies should therefore assess VEGF kinetics together with markers of the FGF pathway rather than evaluating either pathway in isolation.

A dedicated prospective study evaluating MCT (methotrexate + celecoxib + erlotinib combination) in platinum-refractory oral cavity squamous cell carcinoma assessed both CECs and CEPs as pharmacodynamic biomarkers [42]. A decline in CECs by day 8 was independently associated with improved OS in multivariable analysis, while higher baseline CEC levels also predicted longer OS. These findings are consistent with the hypothesis that tumors with greater baseline angiogenic activity may derive greater benefit from antiangiogenic MCT. By contrast, CEP levels did not retain independent prognostic significance, challenging earlier preclinical work that had identified CEPs as a principal cellular target of metronomic antiangiogenesis.

Pharmacogenomic evidence adds another layer of complexity. In a translational substudy conducted by Orlandi and colleagues, the VEGF-A +936 single-nucleotide polymorphism was significantly associated with PFS in patients with advanced castration-resistant prostate cancer treated with metronomic cyclophosphamide [44]. This remains the only formal pharmacogenomic analysis focused specifically on MCT sensitivity rather than on response to anti-VEGF antibodies. However, neither this variant nor other VEGF pathway polymorphisms have been prospectively validated in MCT-treated populations.

Angiopoietin-2 (Ang-2), a regulator of vascular remodeling that acts in concert with VEGF, is another biologically plausible but clinically unvalidated MCT-specific candidate. In a preclinical postsurgical adjuvant model, the combination of an ANGPT2-neutralizing antibody with low-dose metronomic chemotherapy suppressed metastatic growth by attenuating both angiogenic and inflammatory endothelial responses within the metastatic niche, an effect not achieved by either intervention alone [45]. Although circulating Ang-2 has established prognostic and predictive relevance in bevacizumab-treated gastric and colorectal cancer, no clinical study has yet examined baseline or on-treatment Ang-2 specifically as a predictor of MCT response. This represents a clear opportunity for correlative analyses in existing MCT cohorts.

Cancer-associated fibroblasts (CAFs), a major stromal component of desmoplastic tumors such as breast and pancreatic cancer, may also contribute to schedule-dependent differences in treatment response. In a gastric cancer xenograft model, both CAF abundance and intratumoral VEGF levels were significantly lower after low-dose metronomic capecitabine or 5-fluorouracil than after conventional MTD dosing [50]. Metronomic treatment also did not increase the chemoresistance markers GSTP or MDR1, in contrast to the CAF-associated resistance phenotype observed with conventional schedules. These findings suggest that the antiangiogenic effects of MCT may be accompanied by stromal remodeling and reduced CAF-mediated resistance, though this remains a preclinical observation.

Taken together, the inconsistent performance of individual angiogenic biomarkers across these studies likely reflects several overlapping factors. First, redundancy within angiogenic signaling pathways means that VEGF measurement alone may fail to capture compensatory activity from FGF-2, angiopoietins, or other proangiogenic factors, as illustrated by the discordant VEGF and FGF-2/IL-8 findings between the MOVE and Hellenic Cooperative Oncology Group studies [29,54]. Second, tumors may engage compensatory signaling upon sustained antiangiogenic pressure, shifting the relevant biomarker from a static baseline measurement to a dynamic, on-treatment one, as suggested by the divergent baseline and on-treatment VEGF-A patterns observed in the Calleri and MOVE cohorts [29,41]. Third, most angiogenic biomarkers have been assessed at a single static timepoint, whereas the biology they aim to capture is inherently time-dependent, particularly in the metronomic setting, where drug exposure is continuous rather than pulsed. These considerations argue for multiplexed, longitudinal biomarker panels rather than single-analyte, baseline-only approaches in future MCT trials.

### 4.2. Immune Biomarkers

The immunomodulatory component of MCT, particularly the depletion of regulatory T cells, gives rise to a separate set of candidate biomarkers based on immune-cell composition in both tumor tissue and peripheral blood. CD4+CD25+FoxP3+ Tregs are the principal cellular targets of low-dose metronomic cyclophosphamide. Their pretreatment abundance and the kinetics of their depletion during therapy are therefore biologically plausible predictive markers [19].

Peripheral blood immune profiling has been explored in the Bayesian adaptive trial of MCT combined with anti-PD-1 therapy in metastatic breast cancer [22]. Changes in Treg proportions and CD8+ T-cell activation markers during treatment emerged as potentially measurable correlates of response. This is consistent with preclinical and translational evidence showing that low-dose CTX can reduce the peripheral Treg ratio within days of treatment initiation [19], creating a biological window that may be suitable for early response assessment.

Mass cytometry profiling performed in the Mo et al. trial provided the most detailed characterization to date of immune reprogramming during MCT [22]. Clinical benefit was associated with dynamic changes in both myeloid and lymphoid populations, including reductions in CTLA4+ Treg subsets and CD206+ M2-polarized macrophages, together with shifts in CD33+ myeloid cell frequency. These findings are consistent with coordinated remodeling of an immunosuppressive microenvironment during MCT–ICI treatment. Importantly, none of the baseline immune variables examined, including PD-L1 expression and tumor mutational burden, predicted benefit. This suggests that dynamic on-treatment immune changes may be more informative than static baseline profiling in the context of MCT–ICI combinations.

Broader inflammatory indices, including the neutrophil-to-lymphocyte ratio, platelet-to-lymphocyte ratio, and myeloid-derived suppressor cell frequency, have also been proposed as surrogate biomarkers of MCT response, particularly when MCT is combined with ICIs. Although NLR and PLR are well-established prognostic markers across multiple tumor types and therapeutic settings, neither has been specifically evaluated as a predictive marker of MCT benefit in a dedicated prospective or retrospective study. An indirect mechanistic link nevertheless exists, as elevated PLR has been associated with higher intratumoral Treg density in TNBC [55]. Whether baseline NLR or PLR can identify patients most likely to benefit from MCT-induced immune remodeling remains biologically plausible but untested.

Tumor-infiltrating lymphocyte density, an established predictor of response to conventional neoadjuvant chemotherapy in TNBC, has not been formally assessed as a biomarker of MCT response. Tumors with high TIL but disproportionate Treg infiltration may be most susceptible to metronomic immune modulation, whereas immune-cold tumors may not be sufficiently primed by MCT alone.

Myeloid-derived suppressor cells are another immunosuppressive population mechanistically linked to MCT. Preclinical studies consistently show that metronomic cyclophosphamide, gemcitabine, and capecitabine reduce circulating and intratumoral MDSCs and attenuate MDSC-mediated immune suppression, in some cases more effectively at low metronomic doses than at higher doses of the same agents [46]. In glioblastoma, metronomic capecitabine has also been associated with lower circulating MDSC levels and increased infiltration of cytotoxic immune cells into tumor tissue [47]. No prospective study has yet examined baseline MDSC levels or on-treatment MDSC kinetics as predictors of MCT response. For now, MDSC measurements should therefore be viewed as pharmacodynamic rather than validated predictive biomarkers.

A further barrier to the clinical translation of these immune biomarkers is practical rather than biological. Treg, CD8:Treg, and MDSC quantification currently rely on flow- or mass-cytometry panels that vary across laboratories in antibody clones, gating strategies, and reference thresholds, precluding direct comparison between studies. The timing of blood collection relative to treatment cycles is similarly inconsistent, despite evidence that these populations shift rapidly. Treg depletion can occur within days of MCT initiation [19]. Until standardized, validated assay protocols and collection-timing windows are established and shown to be reproducible across centers, immune biomarkers of MCT response are likely to remain research tools rather than clinically actionable tests.

### 4.3. Tumor Proliferative Index: The Ki67 Paradox

Ki67 occupies a paradoxical position in MCT biomarker research. Its predictive value in conventional MTD chemotherapy is well established, as higher Ki67 levels reflect greater sensitivity to replication-dependent cytotoxic agents and underlie its use as a stratification marker in luminal breast cancer. Whether the same relationship applies to MCT has not been tested in adequately powered prospective studies.

Mechanistically, several arguments suggest that Ki67 is more likely to be non-predictive or even negatively associated with MCT efficacy. The principal cellular targets of MCT, including endothelial cells and Tregs, do not express Ki67 in a manner that necessarily differs between highly and poorly proliferative tumors. The antiangiogenic activity of MCT is determined by vascular dependence rather than by the proliferative index of malignant cells, while its immune effects depend more closely on Treg abundance and the broader immune phenotype. In addition, slowly proliferating tumors with low Ki67 may be more likely to harbor immunosuppressive microenvironments, potentially making them better candidates for MCT-mediated immune reprogramming and antiangiogenic targeting.

Clinical evidence consistent with this hypothesis comes from an exploratory analysis of a prospective cohort of 72 patients with treatment-refractory ER-positive/HER2-negative metastatic breast cancer treated at our institution with the FulVEC metronomic chemoendocrine regimen, comprising fulvestrant, vinorelbine, cyclophosphamide, and capecitabine. These are preliminary, single-institution findings that will require confirmation in a larger, prospectively followed FulVEC cohort (Wysocki et al., unpublished data). In this exploratory analysis, Ki67 was not a predictive factor for ORR, PFS, or OS at any of the prespecified cutoffs examined, including 20%, 30%, 40%, and 50%. This contrasts with the established predictive role of Ki67 in the same disease subtype treated with endocrine therapy alone and suggests that the addition of MCT changes the biological determinants of treatment response. These findings support the view that Ki67 should not be used as a selection biomarker for MCT and that its lack of predictive value may represent a mechanistically coherent phenomenon rather than a statistical artifact.

### 4.4. Molecular Biomarkers and Liquid Biopsy

#### 4.4.1. FOXC1

FOXC1 is the most advanced molecular biomarker candidate to emerge from a phase III MCT trial. This forkhead box transcription factor is associated with basal-like TNBC biology, epithelial–mesenchymal transition, and tumor angiogenesis. In a post hoc exploratory immunohistochemical analysis of baseline samples from SYSUCC-001, FOXC1 was assessed in 338 patients, corresponding to approximately 78% of the trial population. FOXC1-high tumors, observed in 149 patients, derived substantial benefit from metronomic capecitabine, with a DFS HR of 0.33 (95% CI 0.16–0.68; *p* = 0.0027) and an OS HR of 0.25 (95% CI 0.10–0.62; *p* = 0.0028). By contrast, FOXC1-low tumors showed no benefit and a numerical trend toward worse outcomes, with a DFS HR of 1.32 (95% CI 0.75–2.33; *p* = 0.34) and an OS HR of 1.39 (95% CI 0.74–2.60; *p* = 0.31).

A prespecified prospective–retrospective correlational analysis of the GEICAM/CIBOMA phase III trial evaluating conventionally dosed capecitabine in patients with TNBC yielded the opposite result. Using the standardized Veresca FOXC1 immunohistochemical assay developed by Onconostic Technologies, FOXC1-low, non-basal-like tumors independently predicted benefit from adjuvant capecitabine, with distant relapse-free survival HRs of 0.41 for Ki67 below 20% and 0.31 for Ki67 of at least 20%, whereas FOXC1-high basal-like tumors showed no advantage [48]. FOXC1-based classification was highly concordant with PAM50 molecular subtyping, supporting its biological validity as a discriminator between basal-like and non-basal-like TNBC.

The predictive direction of FOXC1 in the context of MCT therefore remains unresolved. The discordance between SYSUCC-001 and GEICAM/CIBOMA may reflect important differences in dosing schedules, with continuous metronomic capecitabine in SYSUCC-001 and a conventional 14 days on/7 days off schedule in GEICAM/CIBOMA. Differences in assay standardization are a further non-biological explanation: SYSUCC-001 did not use a standardized commercial FOXC1 assay, whereas GEICAM/CIBOMA employed the Veresca platform, so scoring thresholds are not directly comparable between the two trials. Differences in patient populations may also have contributed. Rather than excluding FOXC1 as a biomarker, these findings suggest that its predictive effect is schedule-dependent and that it may distinguish sensitivity to metronomic versus conventional capecitabine through distinct pharmacological mechanisms. Resolving this question will require prospective validation with a standardized FOXC1 assay in a trial that directly compares metronomic and conventional capecitabine schedules.

#### 4.4.2. ctDNA

Circulating tumor DNA has emerged as a promising liquid-biopsy marker in NSCLC. In the phase II study of metronomic oral vinorelbine combined with cisplatin and concurrent radiotherapy in unresectable stage III disease, ctDNA negativity at baseline or at early on-treatment time points identified patients with substantially longer PFS and OS [30]. It remains unclear whether ctDNA status in this setting primarily reflects tumor burden, genomic complexity, or a biological phenotype specifically associated with sensitivity to MCT.

More broadly, on-treatment ctDNA clearance has been repeatedly associated with favorable outcomes across both targeted therapy and immunotherapy in NSCLC, suggesting that its prognostic value may not be specific to MCT but rather reflects a general marker of treatment efficacy. What remains untested is whether the kinetics of ctDNA clearance during continuous, low-dose exposure differ mechanistically from clearance during pulsed MTD regimens, which would support ctDNA as a genuinely MCT-specific, rather than treatment-agnostic, pharmacodynamic marker.

The feasibility of repeated ctDNA sampling is especially attractive in the context of orally administered metronomic regimens, which are often delivered at home. Serial liquid biopsy may therefore provide a practical alternative to tissue-based biomarker assessment in NSCLC and other tumor types, particularly when only small diagnostic biopsy samples are available.

### 4.5. Pharmacodynamic Cytokine Profiling

In a prospective pharmacodynamic study of 38 heavily pretreated patients with advanced gastrointestinal cancers who received metronomic UFT, cyclophosphamide, and celecoxib, plasma VEGF and soluble VE-cadherin concentrations were significantly higher in patients with progressive disease than in those with stable disease [49]. CD133 expression on peripheral blood mononuclear cells, characterizing endothelial progenitors, also increased only in progressors. These findings identify VEGF, soluble VE-cadherin, and CD133 as potential on-treatment markers of resistance.

The same study also linked 5-FU pharmacokinetic exposure to outcome. An area under the concentration–time curve above 1.313 h × μg/mL and a maximum plasma concentration above 0.501 μg/mL were associated with disease stabilization and longer PFS and OS. These observations provide an early pharmacokinetic rationale for exposure-guided MCT dosing, a concept further considered in the context of FulVEC in Section 4.6. Because of the small sample size and exploratory design, however, these findings should be regarded as hypothesis-generating.

A complementary multiplex cytokine analysis of the Epitopes-CRC01 cohort identified treatment-related increases in IL-6 and IL-8 as pharmacodynamic correlates of response in chemorefractory gastrointestinal cancers [33]. These results extend the biomarker panel beyond angiogenic factors and suggest that immune-related cytokines may also reflect the biological effects of MCT.

In a prospective study comparing metronomic capecitabine with CAPOX as maintenance therapy in mCRC, an increase in FGF-2 during the second cycle relative to baseline was associated with longer PFS, with median values of 12.85 and 7.57 months, respectively [56]. The association did not reach statistical significance in the overall analysis. Whether FGF-2 upregulation reflects a favorable treatment response or the compensatory activation of an alternative angiogenic pathway remains uncertain. If the latter interpretation is correct, FGF-2 may represent an early and potentially targetable mechanism of resistance.

### 4.6. On-Treatment Clinical Biomarkers: Adverse Events as a Surrogate of Pharmacological Exposure

A distinct strategy for MCT biomarker development is to use treatment-related adverse events as indirect indicators of adequate drug exposure. This approach avoids some of the logistical difficulties associated with repeated tissue or blood sampling. The underlying hypothesis is that although metronomic regimens are often administered at fixed low doses, substantial interpatient pharmacokinetic variability may lead to markedly different systemic exposure at the same nominal dose. Patients who experience no adverse events may therefore be pharmacologically underexposed rather than intrinsically resistant.

Clinical support for this concept comes from our prospective cohort of 72 patients with HR-positive/HER2-negative metastatic breast cancer treated with the FulVEC regimen of fulvestrant, vinorelbine, cyclophosphamide, and capecitabine [52]. A consistent monotonic gradient was observed across adverse-event categories. Patients with grade 0 adverse events who required no dose modification had a non-progression rate of 73.2% and a biochemical benefit rate of 68.4%. Corresponding rates were higher among patients with grade 1 adverse events requiring dose reduction (84.2% and 90.9%) and highest among those with grade 2 events leading to treatment delay (91.7% and 100.0%). The correlation between adverse-event grade and treatment duration was statistically significant (Spearman r = 0.258; *p* = 0.043), and the same directional pattern was observed across efficacy outcomes.

These findings suggest that adverse events during MCT may serve as a real-time pharmacodynamic signal of sufficient exposure, whereas their absence may indicate underdosing rather than treatment failure. The multi-agent structure of FulVEC also permits adaptive redistribution of dose intensity between individual drugs when one component causes disproportionate toxicity. This could preserve overall pharmacological pressure while improving tolerability. Prospective evaluation of adverse-event-guided dose optimization is therefore justified and will be incorporated into our planned metronomic cohort studies.

### 4.7. Gut Microbiome

The gut microbiome is increasingly recognized as a determinant of response to both chemotherapy and immunotherapy. Although MCT-specific evidence remains limited, it provides one of the more direct clinical biomarker signals among the emerging candidates discussed in this section.

In a prospective comparison of patients with HER2-negative metastatic breast cancer receiving maintenance capecitabine, those treated with a metronomic schedule had significantly lower gut microbial diversity, as measured by the unweighted UniFrac index, than those receiving conventional dosing [51]. Redundancy analysis also showed partial separation in microbial community structure between the two schedules. Within the same cohort, a higher relative abundance of *Blautia obeum* was associated with longer PFS, whereas a greater abundance of *Slackia* was associated with shorter PFS, thereby identifying specific bacterial taxa as potential markers of benefit.

The biological plausibility of these associations is supported by broader evidence linking the gut microbiome to systemic immune activation during chemotherapy and checkpoint inhibition. Preclinical studies have shown that *Bifidobacterium* abundance is associated with enhanced antitumor immunity and greater responsiveness to checkpoint blockade. In addition, work on prostate cancer has proposed that schedule-dependent effects of MCT on both the host and tumor-associated microbiota may represent an additional, underappreciated mechanism of action [8].

Prospective studies that correlate both baseline and on-treatment microbiome composition with clinical outcomes are therefore warranted. Such studies would be relatively feasible and inexpensive, particularly because most MCT regimens are administered orally in the outpatient or home setting.

Across these seven domains, three candidates currently stand out as the most clinically advanced: (i) FOXC1, the only biomarker to emerge from a phase III trial, although with a direction of effect that is schedule-dependent and still unresolved; (ii) circulating endothelial cell kinetics, supported by concordant findings across independent breast cancer and HNSCC cohorts; and (iii) on-treatment immune or adverse-event dynamics, which consistently outperform static baseline measurements across multiple mechanistic domains. None of these candidates has been prospectively validated, and each will require confirmation in a trial specifically designed and powered for that purpose before it can inform clinical decision-making.

## 5. Discussion

### 5.1. The Evidence Landscape: What Has Been Established

The evidence reviewed here supports a clear conclusion: MCT is an active treatment strategy and, in selected settings, may offer advantages over conventional approaches in breast cancer, HNSCC and NSCLC, while its efficacy as maintenance therapy is established in mCRC. The publication of SYSUCC-001 and MECCA marked a turning point for MCT in breast cancer, moving it beyond an empirical later-line option and providing phase III support in both early-stage and metastatic disease. In NSCLC, TEMPO LUNG remains the only randomized trial and addresses an important clinical need among patients who are not candidates for platinum-based combination chemotherapy, a population with historically limited treatment options.

These studies also share an important methodological limitation that directly affects biomarker interpretation. None was designed, powered, or prospectively specified to identify predictive markers of differential treatment benefit. Consequently, the available biomarker analyses are exploratory, hypothesis-generating, and vulnerable to the statistical limitations inherent in post hoc and unplanned comparisons. Among the candidates identified to date, FOXC1 from SYSUCC-001 is the most advanced. Even so, it should be regarded as a priority for prospective validation rather than as a clinically established predictive tool.

Beyond this trial-level limitation, the biomarker literature reviewed in Section 4 shares three recurring methodological weaknesses. First, most studies are small, frequently enrolling fewer than 100 patients, which limits statistical power to detect all but the largest effect sizes and increases the risk of false-positive findings. Second, a substantial proportion are retrospective or single-arm, precluding a direct, controlled comparison of outcomes between biomarker-positive and biomarker-negative patients. Third, essentially none of the candidates discussed, including FOXC1, circulating endothelial cell kinetics, and on-treatment immune or adverse-event dynamics, has been evaluated in an independent validation cohort. Addressing these three weaknesses through adequately powered, prospectively designed studies is a prerequisite for any candidate biomarker to inform clinical decision-making.

### 5.2. The Mechanistic Rationale for a Distinct Biomarker Profile

A central premise of this review is that MCT should not be viewed simply as a lower-dose form of MTD chemotherapy. It is a mechanistically distinct treatment modality with different biological targets, pharmacodynamic effects, and determinants of response. This distinction has major implications for biomarker development.

The principal targets of MCT include tumor-associated endothelial cells, regulatory T cells, and immunosuppressive myeloid populations rather than malignant cells alone. Predictive biomarkers are therefore likely to reflect the composition and functional state of the tumor microenvironment, including angiogenic dependence, immune contexture, and cytokine signaling. Biomarkers developed for conventional chemotherapy, such as Ki67, DNA repair markers including ERCC1, or pharmacogenomic variants linked to replication-dependent cytotoxicity, cannot be assumed to retain the same relevance in MCT without specific validation.

The Ki67 paradox illustrates this mechanistic divergence. In conventional MTD chemotherapy, high Ki67 identifies a larger proliferating tumor cell fraction and therefore greater vulnerability to replication-dependent cytotoxic agents. In MCT, however, the dominant pharmacological effects are mediated through different cellular compartments and pathways. Ki67 may therefore have limited mechanistic relevance. Its lack of predictive value in the FulVEC cohort and other metronomic series should not necessarily be interpreted as an uninformative negative finding. Rather, it supports the broader hypothesis that MCT response is determined less by tumor cell proliferation than by microenvironmental features. This observation argues for redirecting biomarker development toward angiogenic (including stromal), immune, and pharmacodynamic domains.

### 5.3. Priority Research Directions for Biomarker Validation

The available evidence supports a translational research agenda focused not only on validating individual markers but also on defining a composite biological profile of MCT sensitivity. Such an “MCT-sensitive phenotype” could enable the prospective identification of patients most likely to derive meaningful benefit across tumor types and clinical settings.

The mechanistic framework outlined in this review suggests that an MCT-responsive tumor may be characterized by high angiogenic dependence, reflected by elevated baseline VEGF and CEC levels, together with an immunosuppressive microenvironment marked by increased Treg infiltration and an unfavorable CD8 ratio. In TNBC, FOXC1-high status may represent an additional component of this phenotype. By contrast, immunologically active tumors with high TIL density, low Treg content, and a high proliferative index—features often associated with greater sensitivity to MTD chemotherapy—may derive less incremental benefit from the antiangiogenic and immunomodulatory effects of MCT. This hypothesis, however, requires prospective testing in biomarker-stratified trials.

Four research priorities emerge from the current evidence.

First, FOXC1 should be prospectively validated in a biomarker-stratified adjuvant MCT trial in TNBC using a standardized immunohistochemical assay and predefined thresholds.

Second, future phase II MCT studies should incorporate dedicated pharmacodynamic analyses of CEC kinetics at prespecified time points, such as days 0, 8, 30, and 60. This would help determine whether an early decline in CECs predicts long-term outcome and whether CEC dynamics can contribute to biological dose optimization.

Third, upcoming MCT–ICI trials should include composite peripheral blood immune profiling as a major translational endpoint. This should encompass baseline and on-treatment assessment of the Treg ratio, MDSC frequency, and PD-1-positive CD8-positive T-cell dynamics.

Fourth, adverse-event-guided dose optimization should be prospectively evaluated as a pragmatic surrogate of adequate pharmacological exposure in multi-agent metronomic regimens.

Together, these priorities define a pathway toward a biologically informed and patient-selection-driven model of MCT development. Future studies should evaluate these domains as part of an integrated multiplex panel rather than as isolated endpoints. Such an approach may help identify the composite phenotype associated with MCT sensitivity and determine which combination of biological features best predicts differential benefit from metronomic versus conventional chemotherapy.

### 5.4. Clinical Context: Who Should Receive MCT and How Should They Be Selected?

In the absence of validated predictive biomarkers, selection for MCT must currently rely on disease characteristics, treatment setting, and patient-related factors. Based on the evidence reviewed, the strongest clinical indications include: (1) early TNBC after completion of standard adjuvant chemotherapy, as evaluated in SYSUCC-001; (2) HR-positive/HER2-negative metastatic breast cancer in patients who cannot receive CDK4/6 inhibitors or who require chemotherapy, as supported by MECCA and METEORA-II; (3) advanced NSCLC in patients considered unfit for platinum-based combination therapy, including elderly individuals and those with performance status 2, as shown in TEMPO LUNG and MOVE; and (4) maintenance treatment in mCRC following CAPOX-bevacizumab induction, as established in CAIRO3.

The recurrent observation that MCT is feasible and clinically active in elderly, frail, and performance-status-2 populations is particularly important. This pattern has been observed across tumor types, including the VICTOR-6 real-world cohort in breast cancer, TEMPO LUNG, and the individual patient data meta-analysis in NSCLC. The biological basis of this apparent susceptibility remains uncertain. It may reflect differences in tumor biology, altered pharmacokinetics at low continuous doses, or age-related changes in antitumor immunity and the tumor microenvironment. These possibilities warrant dedicated translational investigation.

## 6. Conclusions

Metronomic chemotherapy has evolved from an experimental concept into an evidence-based clinical strategy in breast cancer, NSCLC, mCRC, and, more recently, head and neck squamous cell carcinoma, as demonstrated by the phase III TMC-I trial. Its biological distinction from conventional cytotoxic chemotherapy—arising from antiangiogenic, immunomodulatory, and cytostatic effects rather than predominantly replication-dependent tumor cell killing—has several important clinical implications.

First, MCT should not be regarded solely as a palliative alternative for patients who are unable to tolerate standard chemotherapy. Instead, it should be considered a mechanistically distinct therapeutic approach that may offer particular benefit in selected biological and clinical contexts. Second, biomarker development for MCT should proceed independently of the frameworks established for MTD chemotherapy, as the relevant determinants of response are likely to arise from angiogenic, immune, stromal, and pharmacodynamic features rather than tumor cell proliferation alone. Third, the positive findings from SYSUCC-001, MECCA, METEORA-II, TEMPO LUNG, and TMC-I provide a sufficiently robust clinical foundation to justify the next generation of biomarker-enriched trials.

Among the available candidates, FOXC1 expression, CEC kinetics, the peripheral Treg:CD8 ratio, and VEGF-A dynamics currently have the strongest combination of biological plausibility and prospective clinical support. None, however, has yet undergone prospective validation in a confirmatory trial. Addressing this validation gap is therefore the central translational priority for the further development of metronomic chemotherapy.

## Figures and Tables

**Table 1:** Key Randomized Trials of Metronomic Chemotherapy in Breast Cancer, NSCLC, mCRC, and Head and Neck Cancer.

Trial	Tumor Type	MCT Regimen	Phase/N	Primary Endpoint	Key Result	Biomarker Data
SYSUCC-001 [20,21]	TNBC (early)	Metronomic cape 650 mg/m^2^ bid × 12 months	III/N = 434	DFS	DFS HR = 0.63 (*p* = 0.027); durable at 10 yr follow-up	FOXC1 (IHC, exploratory, 2025)
MECCA [23]	HR+/HER2− MBC (1L)	Metronomic cape + AI	III/N = 263	PFS	Significant PFS and OS benefit vs. AI alone	None reported
METEORA-II [24]	ER+/HER2− MBC	VEX (oral VNR + CTX + cape)	II rand./N = 140	TTF	TTF 8.3 vs. 5.7 mo; PFS 11.1 vs. 6.9 mo vs. paclitaxel	None
Mo et al. 2024 (Nat Med) [22]	HER2(-) MBC	Metronomic + anti-PD-1	II rand. Bayesian	ORR	MCT+PD-1 clinically effective; immune correlates identified	On-treatment Treg and CD8+ dynamics
TEMPO LUNG [28]	NSCLC (platinum-unfit)	Metronomic VNR 50 mg 3× /wk	II rand./N = 167	G4PFS	G4PFS HR = 0.63 (*p* = 0.006), reduced toxicity	None prospective
MOVE [29]	NSCLC (elderly ≥ 70)	Metronomic VNR 50 mg 3× /wk	II prosp./N = 43	ORR, CB	mOS 9 mo; mTTP 5 mo; well tolerated	VEGF, TSP-1 (non-responders: low baseline VEGF, rapid rise)
Provencio et al. 2021 [30]	NSCLC stage III	Metronomic VNR + cisplatin + RT	II prosp./N = 65	Safety/efficacy	Similar efficacy and lower toxicity vs. standard; ctDNA-neg superior outcome	ctDNA (pilot)
CAIRO3 [32]	mCRC	Cape + bevacizumab maintenance after CAPOX-B	III/N = 558	2nd PFS	Significant 2nd PFS benefit; no QoL compromise	None
Epitopes-CRC01 [33]	mCRC (refractory)	Cape + CTX + aspirin (multimodal MCT)	II prosp. (translational)/N = 77	TME biomarkers	mPFS 2.8 mo; TME immune/angiogenic profiling ongoing	Comprehensive TME panel (plasma + PBMC)
TMC-I (Shah et al. 2026) [36]	HNSCC (platinum-sensitive, recurrent/metastatic)	Metronomic MTX + celecoxib + erlotinib + ultra-low-dose nivolumab	III/N = 422	OS	mOS 10.3 vs. 6.2 mo (HR = 0.57, *p* < 0.0001); 43% reduction in the risk of death	None reported

Abbreviations: AI, aromatase inhibitor; cape, capecitabine; CB, clinical benefit; CTX, cyclophosphamide; DFS, disease-free survival; G4PFS, PFS without grade ≥4 toxicity; MBC, metastatic breast cancer; MCT, metronomic chemotherapy; mo, months; mPFS, median PFS; mOS, median OS; ORR, objective response rate; PFS, progression-free survival; prosp., prospective; rand., randomized; RT, radiotherapy; TNBC, triple-negative breast cancer; TTF, time to treatment failure; VNR, vinorelbine.

**Table 2:** Summary of Candidate Predictive Biomarkers of Metronomic Chemotherapy Response.

Domain	Biomarker	Tumor Type(s)	Best Evidence	Direction	Level of Evidence/Limitations
Angiogenic [29,41]	VEGF-A (serum)	Breast and NSCLC	On-tx decrease in LTRs (BC cohort, N = 46); rise in non-responders (NSCLC MOVE)	Low on-tx VEGF → better outcome	Exploratory; small N; not validated
Angiogenic [41,42]	CECs (baseline)	Breast, Head and Neck	Baseline CEC > Q1 → longer TTP (BC, N = 46); CEC decline at D8 → better OS (HNSCC, N = 91)	High baseline CEC → benefit	Prospective cohorts; no RCT validation
Angiogenic [42,43]	CEPs	CRC and NSCLC	Sequential CEP measurement potentially predictive in colon cancer (preclinical murine model) [43]; not independently prognostic in HNSCC	Conflicting	Small pilot studies only
Angiogenic [29,41]	TSP-1	Breast and NSCLC	Increases during MCT (BC); no change in NSCLC	Inconclusive	Mechanistic signal; weak clinical data
Angiogenic [44]	VEGF-A SNP (+936)	CRPC	Significant predictor of PFS with metronomic CTX	Specific allele → better PFS	Single cohort; not replicated
Angiogenic [45]	Angiopoietin-2 (Ang-2)	Preclinical (mixed models)	Anti-ANGPT2 antibody + LDM chemotherapy limited metastatic growth in a postsurgical adjuvant model	Mechanistic rationale only; no MCT-specific predictive direction established	Preclinical (mouse) only; no clinical biomarker correlate
Immune [46,47]	MDSCs	Preclinical (multiple); GBM (clinical)	LDM CTX/gemcitabine/capecitabine reduce MDSC preclinically; metronomic capecitabine was associated with reduced circulating MDSC in patients with glioblastoma	MDSC reduction → hypothesized benefit (not tested predictively)	Pharmacodynamic signal only; no prospective predictive validation
Immune [22]	Treg (CD4+CD25+ FoxP3+)	Breast	On-tx Treg depletion in an MCT+PD-1 Bayesian trial	Treg depletion → better response	Exploratory within a single RCT
Immune	NLR/PLR	Multiple	Proposed; data for MCT specifically absent	Low NLR → better response (hypothesized)	Hypothesis only; no prospective MCT data
Immune	CD8:Treg ratio	Multiple	Validated for ICI and anti-VEGF (mCRC); extrapolated to MCT	High CD8:Treg → benefit	Indirect; no MCT-specific validation
Proliferative	Ki67	Breast (HR+)	Consistently showed no predictive value in FulVEC cohort (N = 72) and exploratory BC studies	Not predictive of MCT benefit	Mechanistically expected null result; requires formal prospective testing
Molecular [21,48]	FOXC1	TNBC	Post hoc IHC in SYSUCC-001 10 yr update	Schedule-dependent: FOXC1-high → benefit with metronomic cape (SYSUCC-001); FOXC1-low → benefit with conventional cape (GEICAM/CIBOMA)	Two phase III trials; directional discordance—schedule-specific validation required
Molecular [30]	ctDNA	NSCLC	Pilot in metronomic VNR + CDDP + RT (N = 65)	ctDNA-negative → superior outcome	Single pilot study; underpowered
Cytokine [49]	FGF-2 kinetics	mCRC and GI	On-tx FGF-2 increase → longer PFS (SENTRAL/FOLFIRI-bev data)	FGF-2 rise → better PFS (mechanism unclear)	Indirect; not MCT-specific
Cytokine [32]	IL-6, IL-8	mCRC and GI	On-treatment increases in IL-6 and IL-8 associated with MCT response (Epitopes-CRC01; N = 77)	On-tx rise → better outcome	First MCT-specific prospective cytokine data; single cohort; hypothesis-generating
Stromal [50]	CAFs	Gastric (preclinical)	LDM capecitabine/5-FU reduced CAF numbers and intratumoral VEGF versus MTD dosing in gastric cancer xenografts, without increased chemoresistance markers	CAF reduction → hypothesized reduction in chemoresistance	Preclinical xenograft only; no clinical biomarker correlate
Microbiome [51]	Gut microbiota (*Blautia obeum*, *Slackia*)	Breast (HER2-negative mBC)	Metronomic and conventional capecitabine differed in gut microbial diversity; higher *Blautia obeum* → longer PFS, higher *Slackia* → shorter PFS; N = 31	Taxon-specific: *Blautia obeum* favorable, whereas *Slackia* unfavorable	Single small cohort; hypothesis-generating; first MCT-specific microbiome predictive signal
On-treatment clinical [52]	AE grade (adverse events)	Breast (HR+/HER2−)	FulVEC cohort N = 72 (Wysocki et al., Cancers 2026): monotonic AE grade 0 → 2 efficacy gradient; Spearman r = 0.258, *p* = 0.043	Higher AE grade → better outcome (pharmacological under-dosing hypothesis)	Single prospective cohort; hypothesis-generating; requires prospective validation

Abbreviations: Ang-2, angiopoietin-2; BC, breast cancer; CAF, cancer-associated fibroblast; CEC, circulating endothelial cell; CEP, circulating endothelial progenitor; CRC, colorectal cancer; CRPC, castration-resistant prostate cancer; CTX, cyclophosphamide; FGF-2, fibroblast growth factor-2; GI, gastrointestinal; HNSCC, head and neck squamous cell carcinoma; ICI, immune checkpoint inhibitor; LB, liquid biopsy; LTR, long-term responder; MDSC, myeloid-derived suppressor cell; MCT, metronomic chemotherapy; MTX, methotrexate; NLR, neutrophil-to-lymphocyte ratio; NSCLC, non-small cell lung cancer; PD, pharmacodynamic; PLR, platelet-to-lymphocyte ratio; RCT, randomized controlled trial; SNP, single-nucleotide polymorphism; TNBC, triple-negative breast cancer; Treg, regulatory T cell; TSP-1, thrombospondin-1; VNR, vinorelbine.

## Data Availability

No new data were created or analyzed in this study. Data sharing is not applicable to this article.

## Abbreviations

The following abbreviations are used in this manuscript:

5-FU	5-fluorouracil
AE	Adverse event
AI	Aromatase inhibitor
Ang-2	Angiopoietin-2
BC	Breast cancer
CAF	Cancer-associated fibroblast
CB	Clinical benefit
CBR	Clinical benefit rate
ccRCC	Clear cell renal cell carcinoma
CEC	Circulating endothelial cell
CEP	Circulating endothelial progenitor cell
CI	Confidence interval
CRPC	Castration-resistant prostate cancer
CTLA-4	Cytotoxic T-lymphocyte-associated protein 4
CTX	Cyclophosphamide
ctDNA	Circulating tumor DNA
CyTOF	Cytometry by time-of-flight (mass cytometry)
CyTo	Topotecan plus cyclophosphamide regimen
DCR	Disease control rate
DFS	Disease-free survival
ER	Estrogen receptor
FGF-2	Fibroblast growth factor-2
FOXC1	Forkhead box C1
FulVEC	Fulvestrant, vinorelbine, cyclophosphamide, and capecitabine regimen
G4PFS	Progression-free survival without grade ≥4 toxicity
GBM	Glioblastoma
GI	Gastrointestinal
HCC	Hepatocellular carcinoma
HER2	Human epidermal growth factor receptor 2
HNSCC	Head and neck squamous cell carcinoma
HR	Hazard ratio/hormone receptor (context-dependent)
ICI	Immune checkpoint inhibitor
IHC	Immunohistochemistry
IL-6	Interleukin-6
IL-8	Interleukin-8
IPD	Individual patient data
LB	Liquid biopsy
LDM	Low-dose metronomic
LTR	Long-term responder(s)
mBC	Metastatic breast cancer
mCRC	Metastatic colorectal cancer
MCT	Metronomic chemotherapy
MDSC	Myeloid-derived suppressor cell
MSS/pMMR	Microsatellite-stable/proficient mismatch repair
MTD	Maximum tolerated dose
MTX	Methotrexate
NED	No evidence of disease
NLR	Neutrophil-to-lymphocyte ratio
NSCLC	Non-small-cell lung cancer
ORR	Objective response rate
OS	Overall survival
PD-1	Programmed cell death protein 1
PD-L1	Programmed death-ligand 1
PFS	Progression-free survival
PLR	Platelet-to-lymphocyte ratio
QoL	Quality of life
RCT	Randomized controlled trial
RT	Radiotherapy
SNP	Single-nucleotide polymorphism
TMB	Tumor mutational burden
TME	Tumor microenvironment
TIL	Tumor-infiltrating lymphocyte
TNBC	Triple-negative breast cancer
Treg	Regulatory T cell
TSP-1	Thrombospondin-1
TTF	Time to treatment failure
UFT	Tegafur-uracil
VEGF	Vascular endothelial growth factor
VEGFR2	Vascular endothelial growth factor receptor 2
VEX	Oral metronomic vinorelbine, cyclophosphamide, and capecitabine regimen
VHL	Von Hippel–Lindau (gene/protein)
VNR	Vinorelbine
